# Molecular Analysis of Evolution and Origins of Cultivated Hawthorn (*Crataegus* spp.) and Related Species in China

**DOI:** 10.3389/fpls.2019.00443

**Published:** 2019-04-09

**Authors:** Xiao Du, Xiao Zhang, Haidong Bu, Ticao Zhang, Yongchun Lao, Wenxuan Dong

**Affiliations:** ^1^College of Horticulture, Shenyang Agricultural University, Shenyang, China; ^2^Mudanjiang Branch of Heilongjiang Academy of Agricultural Sciences, Mudanjiang, China; ^3^College of Chinese Material Medica, Yunnan University of Traditional Chinese Medicine, Kunming, China

**Keywords:** hawthorn, *Crataegus*, SLAF-seq, nSSR marker, molecular evolution

## Abstract

Hawthorn is of high economic value owing to its medicinal properties and health benefits. *Crataegus* is a member of the Rosaceae family; the genus has a complicated taxonomic history, and several theories on its origin have been proposed. In this study, 53 accessions from seven *Crataegus* taxa native to China and accessions of exotic *Crataegus* species (two from Europe and one from North America) were analyzed by specific locus amplified fragment sequencing (SLAF-seq). In total, 933,450 single-nucleotide polymorphisms were identified after filtering and used to investigate the species’ genomic evolution. Phylogenetic trees derived from nuclear simple sequence repeats (SSRs) and SLAF-seq data showed the same topology, in which *Crataegus maximowiczii* and *Crataegus sanguineae* formed a closely related cluster that was clearly separated from the cluster composed of *Crataegus hupehensis*, *Crataegus pinnatifida*, *Crataegus pinnatifida* var. *major*, *Crataegus bretschneideri* and *Crataegus scabrifolia*. Phylogenetic and structure analysis indicated that the seven Chinese *Crataegus* taxa had two separate speciation events. Plants that evolved the southwestern route shared the genepool with the European species, whereas plants along the northeastern route shared the genepool with the North American species. TreeMix genetic analysis revealed that *C. bretschneideri* may have a hybrid origin. This study provides valuable information on the origins of Chinese *Crataegus* and suggests an evolutionary model for the main *Crataegus* species that native to China.

## Introduction

The genus *Crataegus* (hawthorn), a member of the Rosaceae family, ranges from small shrubs to trees distributed in Eurasia and America (Phipps et al., 1990). Hawthorns are among the most economically important plant species in China, owing to their pleasant flavor, attractive color, and nutrient-rich fruit (Xu et al., 2016). Hawthorn use in preventive medicine dates to the late 1800s. Hawthorns contain biologically active compounds, such as flavonoids, phenols, and oligomeric procyanidins, which have therapeutic benefits (Dickinson et al., 2014; Dahmer and Scott, 2018). Previous laboratory tests and clinical trials have demonstrated the efficacy of hawthorn in the treatment and prevention of cardiovascular disease (Edwards et al., 2012).

On the basis of cladistics analyses of morphological data, Phipps (1990) suggested southwest China and Mexico were ancestral areas for the genus and that trans-Beringian migration of Asian and American *Crataegus* had occurred. *Crataegus* was postulated to have migrated westward from southwest China to Europe, and eastward from East Asia to North America. However, other authors hold conflicting opinions. Evans and Campbell (2002) treated *Crataegus* as subtribe Pyrinae, and based on molecular and non-molecular characters, suggested that *Crataegus* originated in North America. Lo et al. (2009) used sequences for the internal transcribed spacer region, chloroplast DNA regions, LEAFY intron2 to infer relationships among species from eastern Asia, western North America, eastern North America, and Europe; their findings indicated that eastern North America and Europe are probably the most recent common areas of origin for *Crataegus*.

China is the center of *Crataegus* cultivation, and the place of origin of both cultivated and some wild *Crataegus* species. Based on morphological characters, some researchers have proposed that 18 species and six varieties of *Crataegus* are widely distributed across China (Zhao and Feng, 1996; Xin and Zhang, 1997), other authors recognize 20 species of Chinese *Crataegus*, with seven varieties (Dong and Li, 2015). Among these species, *C. hupehensis, C. pinnatifida* var. *major, C. bretschneideri*, and *C. scabrifolia* are cultivated. Previous efforts to understand the phylogenetic and biogeographic history of *Crataegus* have included representatives of Chinese *Crataegus*. Phipps (1990) suggested that *C. scabrifolia* evolved into European *Crataegus* and other Chinese *Crataegus* species (*C. pinnatifida*, *C. hupehensis*, and *C. sanguineae*). Based on sequence data from 14 plastid loci, Zarrei et al. (2015) treated *C. maximowiczii* as section *Sanguineae* and suggested that the origin of the section involved east-to-west trans-Beringian migration from western North America into eastern Asia. Lo et al. (2009) suggested that ancestors of *C. hupehensis*, *C. songorica*, and *C. pinnatifida* dispersed from Europe into Asia. However, a consensus is lacking on the migration direction of Chinese and European *Crataegus*.

Recent studies have examined intraspecific (Wu et al., 2008; Zhang et al., 2008; Sheng et al., 2017) and interspecific relationships (Su et al., 2015; Ma and Lu, 2016) of Chinese *Crataegus*. Previous investigations have used morphological data analyses and limited molecular data, but no study has explored the origin and evolution of cultivated *Crataegus* and related species that are native to China at the genomic level. Plant DNA contains abundant genetic information, and an increasing number of researchers have explored interspecific relationships and diversification of plants using molecular marker information. Molecular markers are used to determine genetic relationships within plant populations with almost 100% reliability (Güney et al., 2018), and random amplified repeats (Erfani-Moghadam et al., 2016; Zarei et al., 2017), inter-simple sequence repeats (Sheng et al., 2017; Emami et al., 2018), and SSRs (Lo et al., 2009; Khiari et al., 2015; Brown et al., 2016) have been widely used for genetic characterization of *Crataegus*, and to analyze genetic diversity among and within accessions of *Crataegus*. Of these molecular markers types, SSR markers have attained considerable popularity in genetic research because they are highly polymorphic, convenient, and codominant. Based on double barcode genotyping systems and sequencing, specific-locus amplified fragment sequencing (SLAF-seq) was developed for *de novo* single nucleotide polymorphism (SNP) discovery and genotyping using reduced representation library sequencing. SLAF-seq is a high-throughput, high-accuracy, and low-cost method with short cycles, and has been used for molecular breeding and analysis of germplasm resources (Huang et al., 2016). SLAF-seq does not depend on a reference genome sequence and is particularly useful for species the lack an assembled reference genome (Sun et al., 2013), because it is possible to perform polymorphism analysis and develop molecular markers directly from the sequence data provided by SLAF-seq (Zheng et al., 2016). Given these advantages, SLAF-seq has been used for rapid mass discovery of SNP markers for polymorphism analysis, system evolution, and germplasm resource identification (Chen et al., 2013; Zhang et al., 2013; Xu et al., 2015).

In the present study, we used SLAF-seq to gain insight into evolutionary relationships among seven *Crataegus* taxa native to China, namely *C. maximowiczii*, *C. sanguineae*, *C. hupehensis*, *C. pinnatifida*, *C. pinnatifida* var. *major*, *C. bretschneideri* and *C. scabrifolia.* These taxa are widely distributed in China and cover most of the different climatic regions in the country. The sampled taxa include the four taxa cultivated in China (*C. hupehensis, C. pinnatifida* var. *major, C. bretschneideri, and C. scabrifolia*) and three species distributed in close proximity to cultivated *Crataegus*. *C. maximowiczii* and *C. sanguineae* are typically distributed in northeastern China. Of the cultivated taxa, *C. pinnatifida* var. *major* is endemic to China and has the longest history in cultivation, and *C. scabrifolia* is considered to be the ancestral *Crataegus* species. *C. pinnatifida* is a species that is widespread throughout China. The seven taxa were selected by other researchers as representatives of Chinese *Crataegus* in previous phylogenetic studies (e.g., *C. pinnatifida*, *C. hupehensis*, and *C. sanguineae*: Phipps, 1990; *C. hupehensis*, *C. sanguineae*, *C. maximowiczii*, and *C. pinnatifida*: Lo et al., 2009; *C. maximowiczii* and *C. sanguineae*: Zarrei et al., 2015). Previous studies provided useful information that partially resolved the phylogenetic history of cultivated *Crataegus* in China. However, the interspecific relationships and evolution of cultivated *Crataegus* in China remain unclear. In the present study, SLAF-seq was used to analyze phylogenetic relationships among 53 accessions of cultivated *Crataegus* and three related species in China based on SSRs and SNPs.

## Materials and Methods

### Plant Material

In total, 53 accessions of Chinese *Crataegus* were sampled, which consisted of *C. maximowiczii* (5 accessions), *C. sanguineae* (4 accessions), *C. hupehensis* (6 accessions), *C. pinnatifida* (14 accessions), *C. pinnatifida* var. *major* (12 accessions), *C. bretschneideri* (10 accessions) and *C. scabrifolia* (2 accessions). The outgroup comprised accessions of three exotic taxa collected from abroad (*C. monogyna* and *C. laevigata* from Europe, and *C. cruss-galli* from North America). The 53 Chinese accessions (Table 1) were broadly distributed across a range of geographic and climatic conditions, and thus were considered to be representative of Chinese hawthorn diversity and to encompass all possible introductory sources in China.

**Table 1:** Details of geographic and sampling information for *Crataegus* investigated in this study.

Taxon	ID	Biogeographic regions	Taxon	ID	Biogeographic regions
*C. bretschneideri*	FLH	Liaoning, China	*C. pinnatifida*	NMGSLH	Inner Mongolia, China
	LNDG	Liaoning, China		WTSSLH	Shanxi, China
	82015	Jilin, China		YB8H	Liaoning, China
	ZF1H	Jilin, China		YB6H	Liaoning, China
	JF1H	Jilin, China		1541SLH	Liaoning, China
	CH	Jilin, China		YR5H	Liaoning, China
	ZF2H	Jilin, China		ZWSLH	Liaoning, China
	555	Jilin, China		GSSZ	Henan, China
	JF2H	Jilin, China		RR5H	Liaoning, China
	FSZ1H	Liaoning, China		RR3H	Liaoning, China
*C. pinnatifida* var. *major*	HG	Shandong, China		HLJMDFSLH	Heilongjiang, China
	XHMZ	Shandong, China		CZSLH	Liaoning, China
	MYDJX	Shandong, China		LH	Liaoning, China
	BRM	Shandong, China		HGSLH	East Asia, Korea
	CK	Shandong, China	*C. maximowiczii*	MSZ1H	Heilongjiang, China
	DMQ	Shandong, China		MSZ2H	Heilongjiang, China
	XLZR	Hebei, China		NASZ	Heilongjiang, China
	JD1H	Beijing, China		MSZ3H	Heilongjiang, China
	DW	Jilin, China		S4	Liaoning, China
	QJX	Liaoning, China	*C. sanguineae*	LNSZ1H	Liaoning, China
	QYMP	Liaoning, China		LNSZ2H	Liaoning, China
	KYRZ	Liaoning, China		LNSZ3H	Liaoning, China
*C. hupehensis*	HBSZ1H	Hubei, China		LNSZ4H	Liaoning, China
	HBSZ2H	Hubei, China	*C. scabrifolia*	YNSZ1H	Yunnan, China
	HBSZ3H	Hubei, China		YNSZ2H	Yunnan, China
	MHL	Shandong, China	*C. monogyna*	DZSZ1H	Britain
	XPZM	Shandong, China	*C. laevigata*	HHSZ	Britain
	TASS	Shandong, China	*C. cruss-galli*	JJSZ	North America

All sampled trees were maintained at the National Hawthorn Germplasm Repository at Shenyang Agricultural University, China (Supplementary Table S1).

### Morphological Measurements

Fresh leaves and fruits were sampled for morphological measurements when mature (from September to early October). Twenty-five leaves and fruits per accession were sampled. The measurement methods followed the technical code for evaluation crop germplasm resources for hawthorn (*Crataegus* L.) (Dong, 2013). Twenty-one characters, including leaf shape, leaf blade lobes, leaf blade margin, and leaf color, were measured. The qualitative trait characteristics and classifications are summarized in Supplementary Table S2.

### DNA Extraction and PCR Amplification

Samples of young leaves were collected, labeled, frozen with liquid nitrogen, and stored at −80°C until DNA extraction. One gram of frozen leaf material was ground for genomic DNA extraction using cetyl-tri-methylammonium bromide in accordance with the protocol of Doyle and Doyle (1990). The DNA quality was checked using a Nanodrop-2000 spectrophotometer (Thermo Fisher Scientific, Wilmington, DE, United States).

Fifty-six samples were analyzed using nuclear SSR (nSSR) markers. PCR amplification was performed in 20 μl reaction mixture consisting of 1 μl template DNA (20–30 ng), 7 μl of 2× Es MasterMix buffer (CWBIO, Beijing, China), 2 μl primers (20 ng/μl), and 10 μl sterile nuclease-free distilled H_2_O. The PCR protocol was as follows: initial denaturation at 94°C for 5 min; 30 cycles at 94°C for 1 min, 56–59°C for 1 min, and 72°C for 1 min; and a final extension at 72°C for 7 min. PCR amplification was carried out in a Thermal Cycler (Applied Biosystems, Foster City, CA, United States). Genotyping was performed using 20 SSR primers (Supplementary Table S3) that were arbitrarily selected from hawthorn transcriptional data (Xu et al., 2016).

The PCR products were separated on a 6% polyacrylamide gel in 0.5× TBE buffer. After electrophoresis, the gel was stained as previously described (Cairns and Murray, 1994). The resulting bands were recorded in a presence–absence matrix in which values represented the presence (1) or absence (0) of a band. Genetic similarities based on Jaccard’s coefficients were calculated using the SIMQUAL program with the Numerical Taxonomy Multivariate Analysis System (NTSYS-PC) v.2.0. The matrix of genetic similarity coefficients was used to generate a dendrogram with the unweighted pair group method with arithmetic mean (UPGMA) method implemented in NTSYS-PC v.2.0 (Rohlf, 1997).

### SLAF-Seq and Analysis

Library preparation and sequencing of SLAF-markers from genomic DNA were performed by the Biomarker Technology Company (Beijing, China). Owing to the lack of a reference genome, the *Malus* genome^1^ (GCA_ 002114115.1) was used for enzyme cutting site prediction. The following criteria were applied to determine the enzyme cutting scheme: (i) the smallest fragments in the repeat sequence, (ii) the fragments are distributed even in the genome, (iii) the length of fragments must be consistent with the scheme, and (iv) the number of SLAFs must meet expectations. After filtering, we used *RsaI* + *HaeIII* (NEB, Ipswich, MA, United States) for digestion, and the predicted target fragment length was 314–344 bp. Sequence data for the control, *Oryza sativa* subsp. *japonica*, was obtained from the Rice Annotation Project Database^2^ for quality control and to ensure the effectiveness of the enzyme cutting scheme. In accordance with the enzyme cutting scheme, the restriction enzymes digested the qualified DNA to obtain the SLAFs; then, the adenine nucleotide (A) was added to the 3′ end of the SLAFs, the dual-index adapter was ligated, and the extract was purified and submitted for pair-end sequencing using an Illumina Hiseq^TM^ 2500 platform (Illumina, Inc., San Diego, CA, United States). Dual-indexing was used to identify the original data from the sequence and obtain reads for the 56 samples. SNPs were called using the Burrows-Wheeler Alignment tool (BWA; Li and Durbin, 2009). Population polymorphism analysis was conducted using high-quality SNPs. Reads with clear index information were clustered based on sequence similarity.

### Phylogenetic Inference and Divergence Time Estimation

A phylogenetic tree was constructed based on the SNPs using maximum likelihood (ML) analyses (Kobert et al., 2016). The ML analyses were performed using RaxML v.8.2.0 (Stamatakis, 2014). Principal component analysis (PCA) was performed using EIGENSOFT (Price et al., 2006). Population structure was investigated using STRUCTURE (Hubisz et al., 2009). To determine the most likely number of ancestral kinships (*K*) in the population, STRUCTURE was run 20 times for each *K* value from 3 to 10. We calculated Δ*K*, which indicates the change in likelihood of different numbers of clusters, and determined the cluster number with the highest Δ*K* value, which represents the most likely number of clusters in the population. We inferred admixture graphs using TreeMix v.1.12 (Pickrell and Pritchard, 2012). Divergence time estimation was conducted using PAML (Stadler and Yang, 2013) based on the SNPs data and fossil age. Fossils for calibration were selected from the TimeTree database^3^. The divergence times between species from the TimeTree database were extracted from all peer-reviewed publications in molecular evolution and phylogenetic that reported estimates of the time of divergence among species. A hierarchical average linkage method was used to estimate divergence times (Ts) of clade pairs to build a Super Time tree, together with a procedure for testing and updating topological partitions to ensure the highest degree of consistency with individual time trees in every study (Hedges et al., 2015).

The split time between *C. pinnatifida*-1541SLH and *C. monogyna*-DZ1H was searched in the database. The estimated divergence data (14.7 Ma) was set as the ancestral node time, which was then used to estimate species divergence times.

## Results

### Plant Morphological Descriptions

**FIGURE 1:**
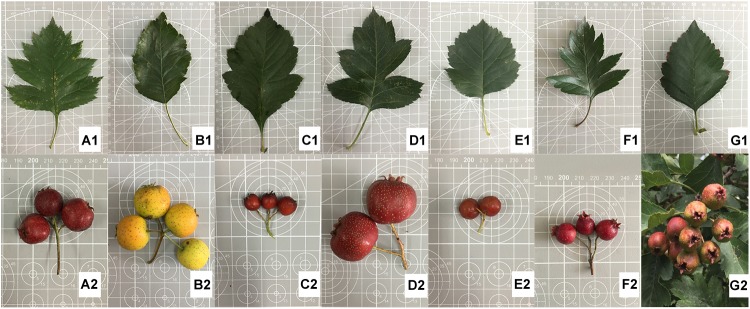
Morphological illustration of the seven *Crataegus* species investigated. **(A)** *C. bretschneideri* – 82015, **(B)** *C. hupehensis* – HB1H, **(C)** *C. maximowiczii* – NASZ, **(D)** *C. pinnatifida* var. *major* – JD1H, **(E)** *C. sanguineae* – LN1H, **(F)** *C. pinnatifida* – HGSLH, **(G)** *C. scabrifolia* – YN1H.

The leaf and fruit characteristics recorded are shown in Supplementary Table S2. The most obvious differences between species were in the leaf blade lobes and fruit size. Based on morphological characteristics (Figure 1), the seven Chinese *Crataegus* taxa were separated into the following four groups: (i) plants with shallowly dissected leaves and small fruit, which contained *C. maximowiczii* and *C. sanguineae;* (ii) plants with moderately lobed leaves and medium-sized fruit, which comprised *C. pinnatifida*; (iii) plants with deeply dissected leaves and large fruit, which consisted of *C. pinnatifida* var. *major* and *C. bretschneideri*; and (iv) plants with non-dissected or shallowly lobed leaves and large fruit, which comprised *C. hupehensis* and *C. scabrifolia.* The *C. maximowiczii* is the only species with have pubescence on the leaf.

### SSR Analysis

The genetic relationships among the *Crataegus* taxa were determined based on a cluster analysis that used loci generated by the nSSR markers. Twenty microsatellite primer pairs were used for the nSSR analysis. Similarity values ranged from 0.49 to 0.96. Genotyping data for 48 alleles were obtained with the 20 primer pairs and used to construct a dendrogram with the UPGMA method (Supplementary Figure S1). The 56 accessions were well separated using the SSR markers.

The nSSR phylogenetic tree showed that *C. monogyna* and *C. laevigata*, which are from Europe, were entirely distinct from the other *Crataegus* species, and *C. cruss-galli*, which is from North America, formed a sister group with *Crataegus* from northeastern China. The seven Chinese *Crataegus* taxa were grouped into two major clusters; one cluster contained *C. maximowiczii* and *C. sanguineae*, whereas the other contained *C. hupehensis*, *C. pinnatifida*, *C. pinnatifida* var. *major, C. bretschneideri*, and *C. scabrifolia*. These two clusters were separated at a similarity value of approximately 0.65. Four accessions (ZWSLH, GSSZ, RR5H, and RR3H) were considered to belong to *C. pinnatifida* based on their morphological characteristics; however, in the nSSR dendrogram, these accessions showed higher similarity to *C. maximowiczii* and *C. sanguineae*.

### SNP Discovery and Population Structure Analysis

In this study, all 56 samples were sequenced and 803,196 SLAF tags were developed in total. The average sequencing depth of the SLAF tag was 12.31-fold, and 164,397 SLAF tags were polymorphic. Fifty-six DNA libraries were sequenced using the SLAF-seq technique, which generated 157.75 M reads, with a mean Q30 of 91.83% and GC content of approximately 41.04% (Table 2).

**Table 2:** Summary statistics for specific-locus amplified fragment sequence data.

ID	Total Reads	GC Percentage (%)	Q30 Percentage (%)	ID	Total Reads	GC Percentage (%)	Q30 Percentage (%)
ZWSLH	2,015,288	43.00	91.34	QYMP	2,607,622	39.89	91.54
HB2H	3,478,596	43.01	91.24	YP8H	2,526,019	40.73	91.94
XLZR	3,275,684	42.78	91.76	YB6H	2,298,033	40.32	92.02
MSZ1H	5,034,166	41.50	91.46	DZ1H	3,439,132	41.17	91.89
82015	2,327,754	41.63	91.21	HB3H	1,741,714	40.70	92.40
HB1H	1,980,132	42.87	91.13	LN2H	2,508,277	40.30	90.76
ZF1H	2,307,880	42.40	91.98	LN4H	2,988,505	40.40	92.14
DW	2,819,383	41.55	91.89	YN1H	2,394,655	40.92	92.31
HHSZ	2,046,640	41.48	92.64	1541SLH	2,322,234	41.15	92.14
GSSZ	2,375,129	42.32	93.06	MSZ3H	3,140,509	39.37	91.97
NASZ	2,433,928	41.10	92.58	LNDG	3,588,855	42.64	91.65
LH	3,055,134	41.50	92.26	YR5H	2,052,058	41.46	92.06
JJSZ	2,177,020	40.35	92.36	WTS2H	1,738,451	40.57	91.59
S4	1,922,386	40.19	92.09	RR5H	1,013,121	41.21	89.55
MSZ2H	2,219,212	39.89	91.80	YN2H	1,760,603	41.91	91.15
BRM	2,609,006	40.20	91.14	HLJMDFSLH	2,239,527	41.56	92.19
TASS	2,761,600	41.84	91.73	RR3H	2,266,488	40.70	91.70
XPZM	3,011,896	40.67	91.72	LN1H	1,891,849	40.02	92.64
JD1H	2,343,060	40.66	92.40	LN3H	2,086,511	40.01	92.57
MHL	1,902,630	41.42	92.32	HG	2,183,162	40.84	92.16
QJX	1,549,868	40.49	91.42	HGSLH	1,874,190	41.08	91.47
JF1H	1,865,336	41.04	91.68	CZSLH	2,491,885	41.74	92.18
FLH	3,754,819	42.96	92.14	JF2H	2,101,713	41.03	91.47
CK	1,242,112	40.12	90.24	FSZ1H	1,884,635	41.40	92.61
CH	3,144,835	39.80	91.62	KYRZ	2,010,776	40.97	91.76
NMGSLH	2,429,365	40.42	92.28	DMQ	1,785,409	40.55	92.04
ZF2H	1,807,502	40.22	91.88	XHMZ	4,670,627	42.87	92.05
555	2,669,796	41.37	91.09	MYDJX	2,632,098	41.92	92.39

The maximum depth sequence of every SLAF tag was accounted for as a reference; using BWA (Li and Durbin, 2009), the reads were aligned to the *Malus* genome, and SNPs were identified with GATK (McKenna et al., 2010) and SAMtools methods (Li et al., 2009). When both methods identified the same SNP, SNP identification was considered to be reliable. Thus, we identified 933,450 SNPs after filtering and used them to investigate genomic evolution among the 56 accessions.

**FIGURE 2:**
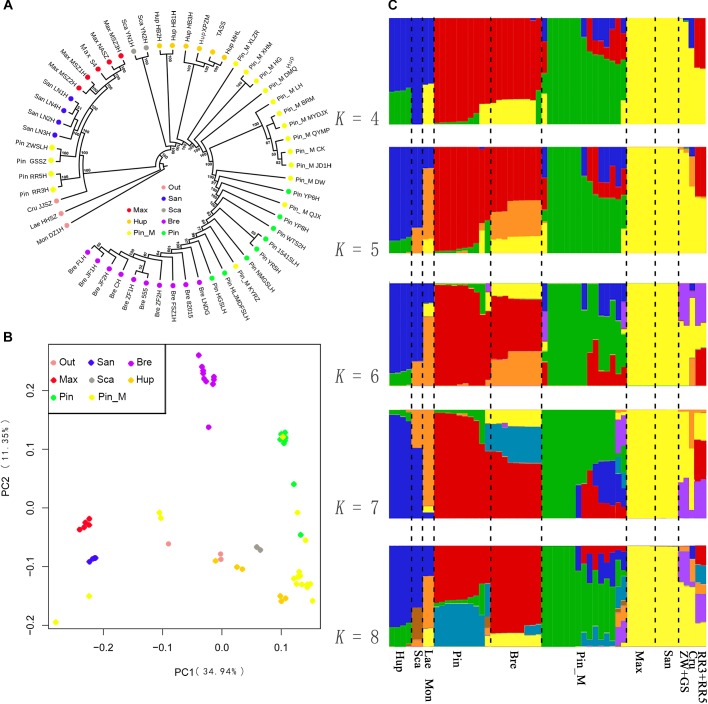
Population structure of 56 samples. **(A)** ML phylogenetic tree constructed using SNPs. Each species group is color coded. **(B)** Principal component analysis (*PCA*) of the 56 samples. **(C)** Bayesian model-based clustering of 56 samples with the number of ancestry kinship (*K*) from 4 to 8. Each vertical bar represents one hawthorn sample. Each color represents one putative ancestral background.

We first constructed a ML phylogenetic tree based on the SNPs data. Given that the morphological characters divided the Chinese *Crataegus* taxa into four groups, based on the molecular data these four groups were predicted to form two clusters. The ML tree (Figure 2A) showed strong evidence for two clusters: cluster A was a northeastern group (*C. maximowiczii* and *C. sanguineae*) that was sister to all other *Crataegus* accessions; cluster B was a widely distributed group, with species ranging from southwestern to central, northern, eastern, and northeastern China (*C. scabrifolia*, *C. hupehensis, C. pinnatifida, C. pinnatifida* var. *major*, and *C. bretschneideri*). In cluster B, accessions of the southwestern Chinese species *C. scabrifolia* was indicated to be an early divergence in the cluster. The central Chinese, species *C. hupehensis* was indicated to be paraphyletic with the northern, eastern, and northeastern species nested within it. These two subclusters were well separated and therefore distantly related. The PCA (Figure 2B) suggested relationships in ordination space that were consistent with topology the A of the phylogenetic tree. *C. maximowiczii* and *C. sanguineae* accessions formed a closely related cluster that was distinctly separated from the cluster comprising the accessions of *C. scabrifolia, C. hupehensis, C. pinnatifida, C. pinnatifida* var. *major*, and *C. bretschneideri*.

To further understand the evolutionary history of Chinese *Crataegus*, we used a Bayesian clustering algorithm with admixed models (Hubisz et al., 2009) to estimate the ancestral proportions for each sample (Figure 2C). The Δ*K* analysis revealed that five populations (*K* = 5) represented the best model for these 56 samples (Supplementary Figure S2). When *K* = 4, the *C. bretschneideri* genepool was indicated to be derived from *C. maximowiczii* (yellow) and *C. pinnatifida* (red); when *K* = 5 to 8, the *C. bretschneideri* genepool predominantly (more than half) was suggested to be derived from *C. maximowiczii* and *C. pinnatifida*. The STRUCTURE analysis (Figure 2C) showed that the southwestern Chinese species *C. scabrifolia* harbored had the genepool from the European species *C. monogyna* and *C. laevigata*, and showed evidence for introgression from *C. scabrifolia* and the two European species. The genepool of the North American species *C. cruss-galli* was similar to that of the northeastern Chinese species *C. sanguineae*. Similar to the SSR dendrogram, the genepool of the accessions ZWSLH, GSSZ, RR5H, and RR3H shared with *C. maximowiczii* and *C. sanguineae*; these findings demonstrated that four accessions did not belong to *C. pinnatifida*. When *K* = 4 to 8, the accessions RR3H and RR5H shared the genepool of *C. pinnatifida* and *C. bretschneideri*. To explore the true identity of these four accessions, we considered them to be “unknown” accessions in the following analysis.

**FIGURE 3:**
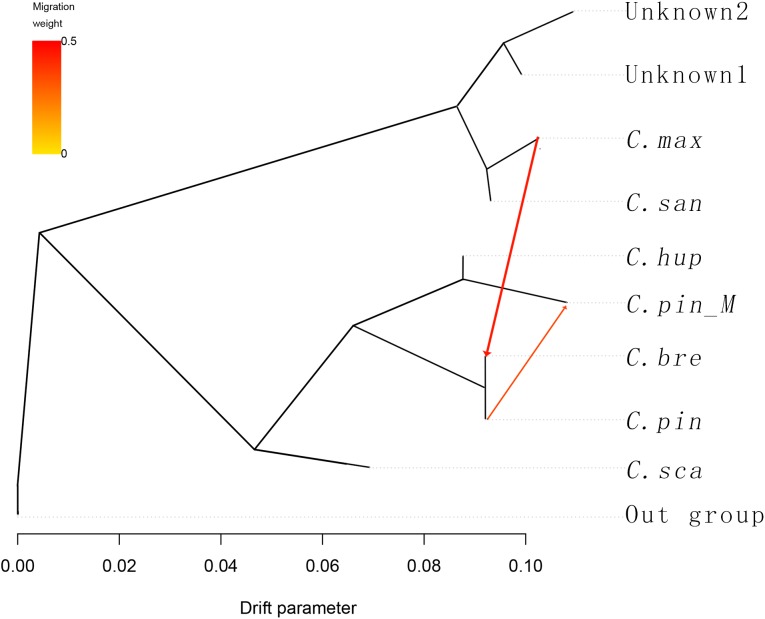
TreeMix analysis of 53 hawthorn samples divided into 9 groups. With *C. cruss-galli*, *C. monogyna* and *C. laevigata* serving as the outgroup population, the arrow corresponds to the direction of migration.

To understand the history of divergence and admixtures, we applied TreeMix to the 10 groups, six species and one variety, and the groups unknown1 (GSSZ and ZWSLH) and unknown2 (RR3H and RR5H). *C. cruss-galli*, *C. monogyna*, and *C. laevigata* were used as the outgroup taxa. TreeMix uses genome-wide allele frequency data and a Gaussian approximation to genetic drift to analyze whether the migration events between species is a pattern of population splits and mixtures in multiple populations. The result contains the population splits and gene flow between species. The arrow in the figure corresponds to the migration events, the darkness of the arrow color indicates the migration edge weight. In the TreeMix result (Figure 3), introgression occurred among *C. hupehensis*, *C. pinnatifida* var. *major*, and *C. pinnatifida*, which indicated that extensive gene flow had occurred in central, northern, and northeastern China. The *C. maximowiczii*, *C. sanguineae*, and two unknown groups formed a cluster that differed markedly from the other groups. Between the two clusters, gene flow from *C. maximowiczii* to *C. bretschneideri* was observed, with an arrow weight of 0.43, which indicated that *C. bretschneideri* is the result of admixture between *C. maximowiczii* and *C. pinnatifida*, with about 43% of the genome derived from *C. maximowiczii*. We did not observe gene flow between the four unknown accessions and other species. Based on the STRUCTURE and TreeMix results, we propose that *C. bretschneideri* may have been of hybrid origin, and shared the genepool with *C. pinnatifida* and *C. maximowiczii*.

### Divergence Time Estimation

**FIGURE 4:**
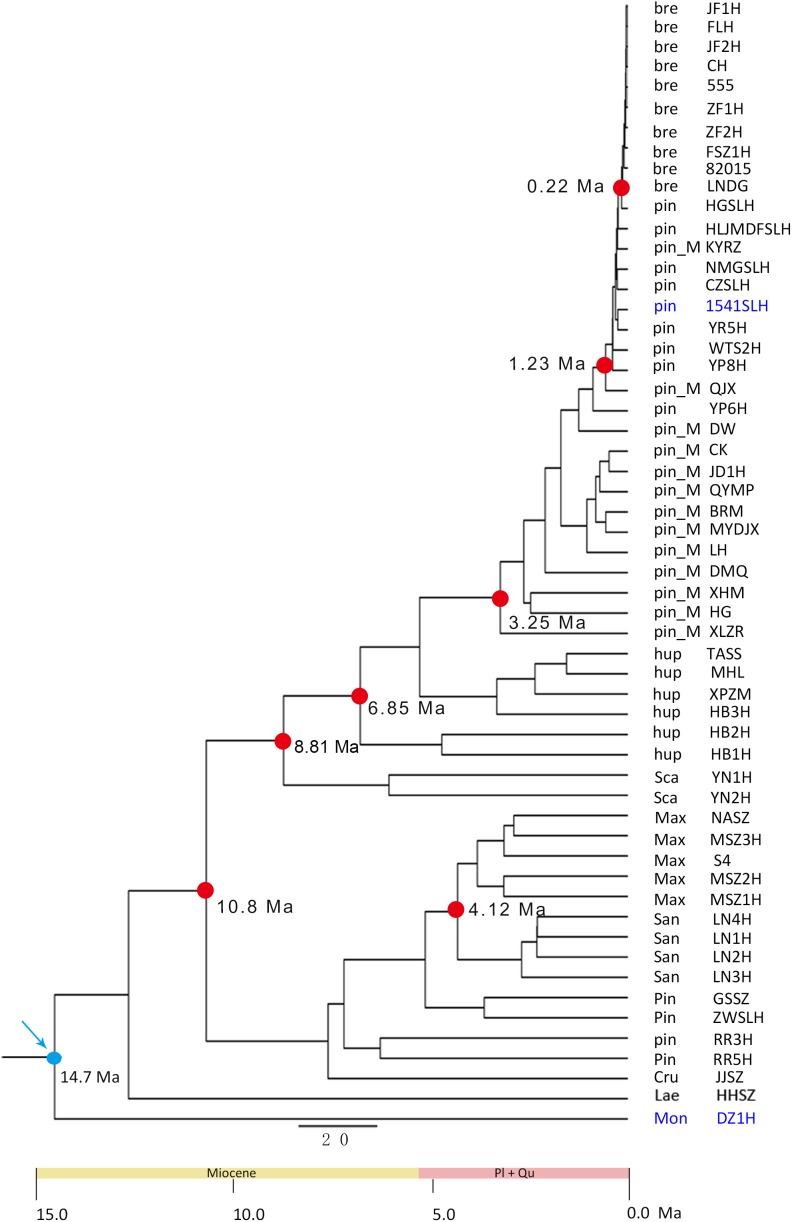
Time-calibrated phylogeny inferred from SLAF-seq data in BEAST V.1.7.5 using node age based on fossil data. Blue arrows designate calibrated node (14.7 Ma). The blue words represent the fossils for calibration. The dots represents the split time.

Based on the SLAF-seq data and fossil calibration, the major diversification events of Chinese *Crataegus* species were estimated to have occurred in the late Miocene and Pliocene (Figure 4). The split between clusters A and B was estimated to be ∼10.8 Ma. The earliest Chinese *Crataegus* to diverge was the southwestern species *C. scabrifolia*, which split at ∼8.81 Ma, followed by the central species *C. hupehensis*, which diverged at ∼6.85 Ma. Subsequent diversification events occurred during the Pliocene. The northeastern species *C. sanguineae* and *C. maximowiczii* split at ∼4.12 Ma; *C. pinnatifida* var. *major*, which is distributed in northern, eastern, and northeastern China, diverged at ∼3.25 Ma; the northern and northeastern species *C. pinnatifida* was diverged at ∼1.23 Ma; and the northeastern species *C. bretschneideri* was the most recent species to diverge and arose at ∼0.22 Ma.

## Discussion

### Phylogenetic Hypothesis for Chinese *Crataegus*

We presented a robust phylogenetic construction for seven Chinese *Crataegus* species based on SLAF-seq data. The ML phylogenetic tree showed evidence for two main clusters. Cluster A includes *C. maximowiczii* and *C. sanguineae*, whereas cluster B comprised *C. hupehensis*, *C. pinnatifida*, *C. pinnatifida* var. *major*, *C. bretschneideri*, and *C. scabrifolia.* From a geographically perspective, cluster A consists of the species in northeastern China, whereas cluster B includes the widespread species that extend from southwestern to northeastern China. Combined the molecular data and the morphological characters, fruit size is an important character for *Crataegus* classification. Consistent with the morphological classification, molecular data showed strong support for the grouping of the two small-fruited species, namely *C. maximowiczii* and *C. sanguineae*. These two species are closely related, and the only differentiating character is pubescence on the leaf of *C. maximowiczii*. In addition to fruit size, leaf dissection is also an important character for discrimination of *Crataegus* species. *C. sanguineae* and *C. maximowiczii* are sympatric with *C. bretschneideri*, *C. pinnatifida*, and *C. pinnatifida* var. *major*, which are distributed in northeastern China. These five species from the northeast were divided into two groups, of which the group that contained *C. bretschneideri*, *C. pinnatifida*, and *C. pinnatifida* var. *major* was more closely related to the southwestern species. The STRUCTURE analysis showed that *C. scabrifolia*, which indicated to be the earliest species to diverge in cluster A, belonged to the same lineage as the European species *C. laevigata* and the North American species *C. cruss-galli*. When *K* = 4 to 7, all *C. scabrifolia* accessions clustered with *C. laevigata* and *C. cruss-galli*.

There are two contrasting perspectives on the migration direction of Chinese and European *Crataegus*. Based on the nSSR dendrogram and STRUCTURE analysis (Figure 2C), we suggest that the seven Chinese hawthorn species may have experienced two different speciation events. The first speciation event occurred in northeastern China, the northeastern species shared the genepool with the North American species. The second speciation event originated in southwestern China and then progressed northward and eastward to northeastern China. The taxa in southwestern route shared genepool with Europe *Crataegus*. The southwestern routed is consistent with Wang’s (1992) description of the plants of other genus and families stretched from the Hengduan Mountains toward the northeast through the Qinling Range, the eastern fringe of the Loess Plateau including the Taihang Range, the Yinshan Range, the Changbai Mountains, and the Xiao Hinggan Mountains of Siberia or the adjacent regions.

Population structure is the result of both present and historical processes, and many factors may change the geographical distributions of plant species (Comes and Kadereit, 1998). Similar to the Quaternary climatic events that caused vegetation changes between southern and northern China (Sun et al., 2000), geological events are critical to the formation and development of the regional flora (Zhe and Jian, 2017; Zhe et al., 2017). In the late Miocene, interspecific divergence events occurred within Chinese *Crataegus* species. Since the late Pliocene, numerous intraspecific differentiation events have occurred. According to previous studies, the third intense uplift of the Qinghai–Tibet Plateau (QTP) and formation of the Hengduan Mountains began at this time (Li and Fang, 1998, 1999; Shi, 1998). The QTP uplift is one of the most important geological events of the Cenozoic; it changed the geography and climate in Asia and led to the Asian monsoon system (Li, 2006; Liu and Dong, 2013). The Asian monsoon system resulted in uneven distribution of precipitation in Yunnan Province and caused the migration of plants (Jacques et al., 2011; Su et al., 2013). Consequently, these events also may have caused the *Crataegus* species in southwestern China to migrate toward the northeast.

In this study, we observed that four accessions were misidentified based on morphological characteristics. The molecular data support the conclusion that these accessions may belong to species other than *C. pinnatifida*; however, additional research is needed to elucidate the true identity of these accessions.

### Hybridization of Chinese *Crataegus* Species

Hybridization is recognized to be an important driving force in plant evolution (Mallet, 2007; Paun et al., 2009) that creates new species, or ecotypes, and results in reticulate evolution (Whitham et al., 1994; Linder and Rieseberg, 2004). Changes in geographical distribution provide opportunities for speciation through hybridization. Hybrids are commonly found in regions where different species overlap. Where species’ ranges overlap, hawthorns show introgressive hybridization, which results in a variety of morphological variants (Talent and Dickinson, 2007). Radford et al. (1968) and Phipps (2015) posited that hybridization is a potential explanatory factor for speciation in *Crataegus*. Furthermore, molecular data have provided evidence for hybridization in *Crataegus* (Lo et al., 2010a,b). *C. bretschneideri* is morphologically very similar to *C. pinnatifida*, and some researchers consider the former to be a variant of the latter species (Dai, 2007). Based on peroxidase isozymograms, Guo and Jiao (1995) suggested that *C. bretschneideri* is closely related to *C. pinnatifida.* On the basis of the present results, we propose that *C. bretschneideri* has a hybrid origin. The STRUCTURE analysis (Figure 2C) indicated that *C. bretschneideri* shared a genepool with *C. pinnatifida* and *C. maximowiczii*; when *K* = 4, the genepool of *C. bretschneideri* was derived from *C. maximowiczii* (yellow) and *C. pinnatifida* (red); when *K* = 5 to 8, the *C. bretschneideri* genepool dominated (more than half) was derived from *C. maximowiczii* and *C. pinnatifida*. The TreeMix results (Figure 3) indicated that gene flow had occurred from *C. maximowiczii* to *C. bretschneideri. C. bretschneideri* is the most recently divergent species, and *C. pinnatifida* was diverged before *C. bretschneideri.* Geographically, *C. bretschneideri* is distributed at the border of *C. maximowiczii* and *C. pinnatifida.* Based on these results, we hypothesize that *C. bretschneideri* arose through hybridization between *C. pinnatifida* and *C. maximowiczii.* The northern species migrated northeast, and hybridization with native species gave rise to new species.

## Conclusion

This study is the first to use SLAF-seq to investigate the evolution and phylogenetic relationships of Chinese *Crataegus*. We hypothesize that the seven *Crataegus* species analyzed in this study experienced two speciation events. The southwestern species, *C. scabrifolia*, was indicated to be the earliest-diverging Chinese species, and shares a genepool with European *Crataegus* species. The northeastern *Crataegus* species share a genepool with the North American species *C. cruss-galli*. Overall, the present results provide valuable information on the origin of *Crataegus* in China.

## Data Availability

The datasets generated for this study can be found in NCBI, SRP151024.

## Author Contributions

XD and WD conceived this project and designed the work. XD, XZ, HB, and YL performed the research. XD and TZ analyzed the data. XD, WD, and XZ wrote the manuscript. All authors contributed critically to the drafts and gave final approval for publication.

## Conflict of Interest Statement

The authors declare that the research was conducted in the absence of any commercial or financial relationships that could be construed as a potential conflict of interest.

## Footnotes

1 https://www.ncbi.nlm.nih.gov/assembly/

2 http://rapdb.dna.affrc.go.jp/

3 http://timetree.org
