# Supplementary material for: Molecular Analysis of Evolution and Origins of Cultivated Hawthorn (Crataegus spp.) and Related Species in China
Source: Front Plant Sci. 2019 Apr 9;10:443. doi: 10.3389/fpls.2019.00443 (PMC6465762; doi:10.3389/fpls.2019.00443)
Supplement: Supplementary file 1 [file Image_1.pdf]

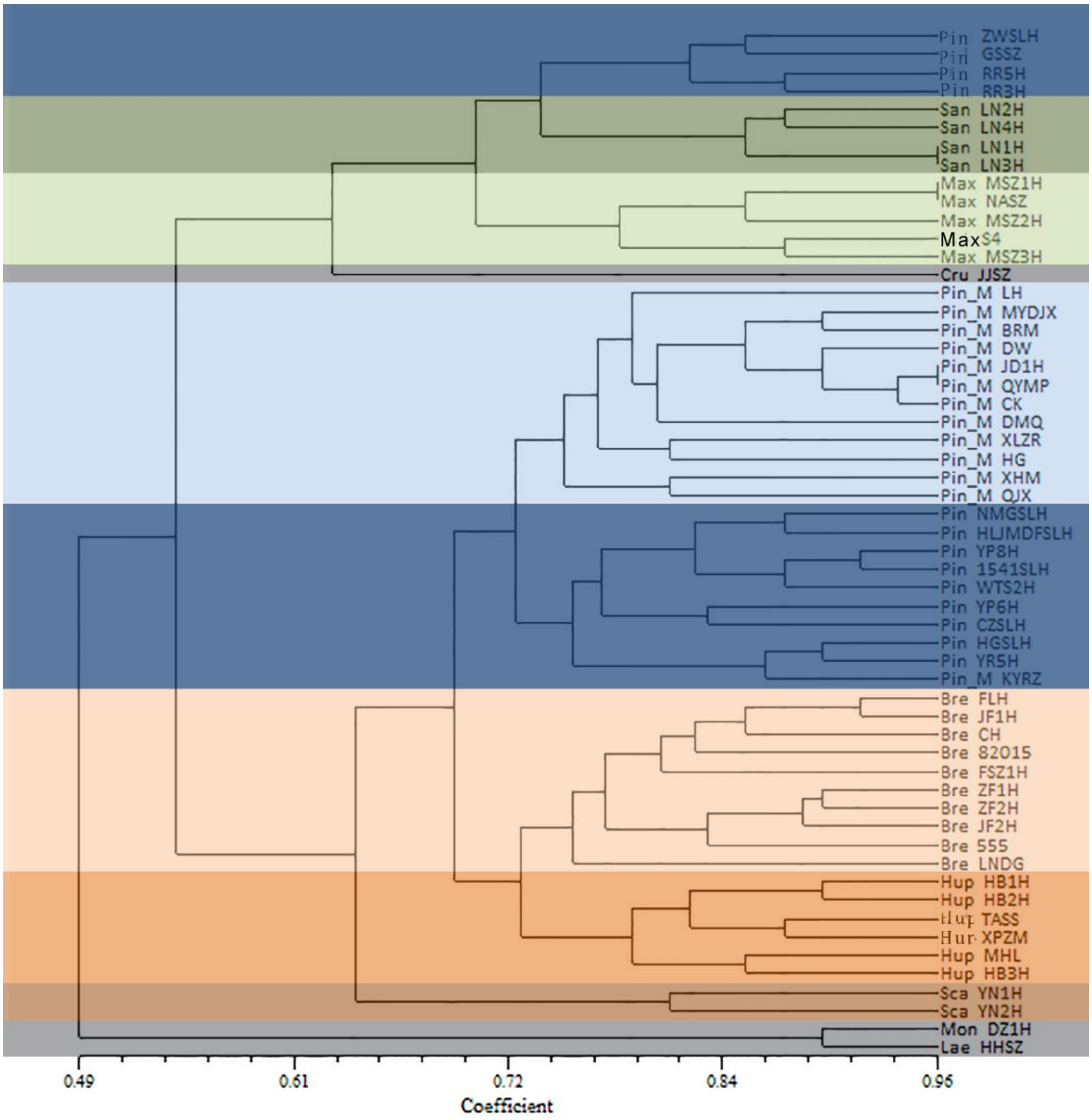

Figure S1 Dendrogram of *Crataegus* species based on nSSR polymorphism. Cluster analysis was performed using the UPGMA method. The names of fifty six individuals are given next to their branches. (Abbreviated species names: San: *C. sanguineae*; Max: *C. maximowiczii*; Cru: *C. cruss-galli*; Pin_M: *C. pinnatifida*. var. *major*; Pin: *C. pinnatifida*; Bre: *C. bretschneideri*; Hup*: C. hupehensis*; Sca: *C. scabrifolia*; Mon: *C. monogyna*; Lae: *C. laevigata.*
