# Supplementary figures and images for: Molecular Analysis of Evolution and Origins of Cultivated Hawthorn (Crataegus spp.) and Related Species in China
Source: Front Plant Sci. 2019 Apr 9;10:443. doi: 10.3389/fpls.2019.00443 (PMC6465762; doi:10.3389/fpls.2019.00443)

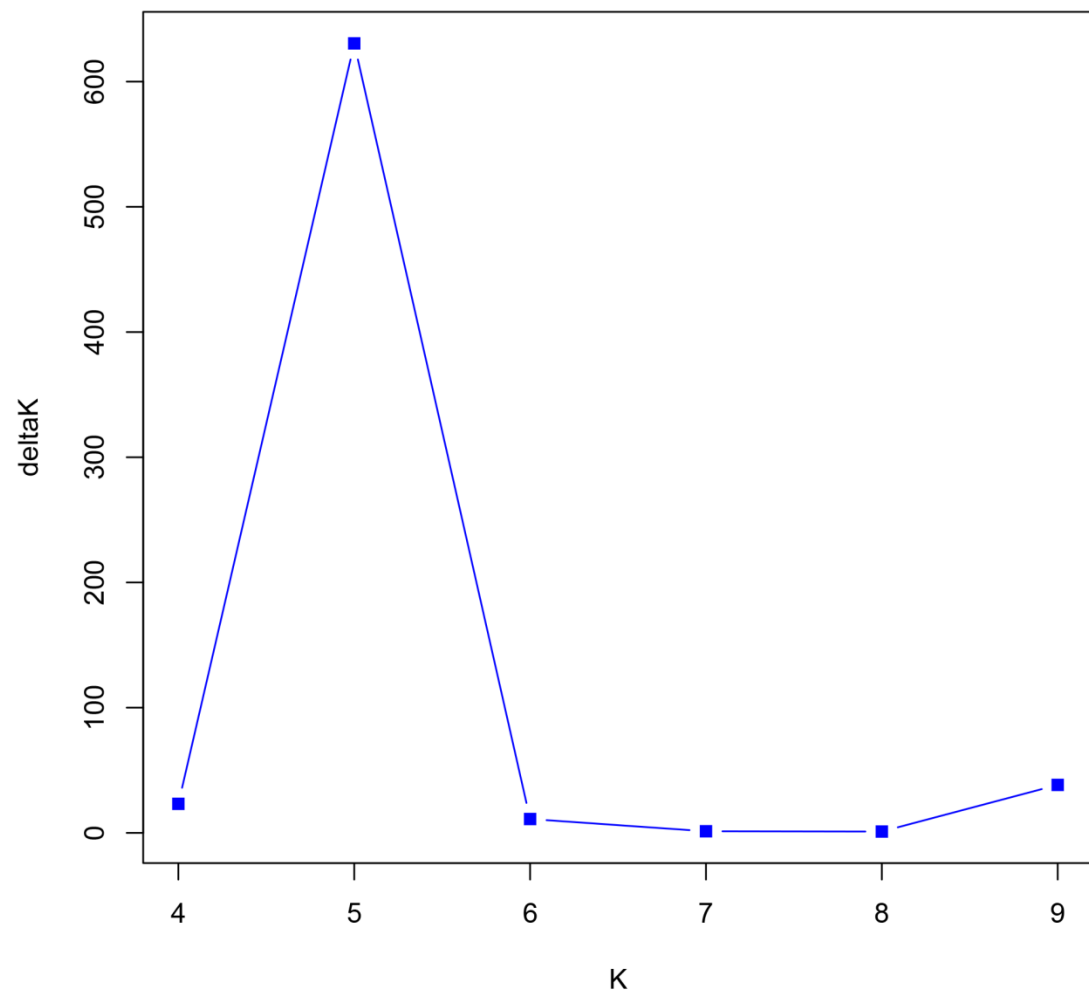

Fig. S2 When  $K=5$ , the  $\Delta K$  is maximum

Supplement: Supplementary file 2 [file Image_2.pdf]
