# Supplementary material for: Molecular Analysis of Evolution and Origins of Cultivated Hawthorn (Crataegus spp.) and Related Species in China
Source: Front Plant Sci. 2019 Apr 9;10:443. doi: 10.3389/fpls.2019.00443 (PMC6465762; doi:10.3389/fpls.2019.00443)
Supplement: Supplementary file 3 [file Table_1.docx]

**Table S1 Collection place and time and the deposited place of *Crataegus* investigated in this study**

| Taxon | ID | Biogeographic regions | Collection Place | Collection Time | Deposited Place |
| --- | --- | --- | --- | --- | --- |
| *C .bretschneideri* | FLH | Northeast, China | Shenyang | 2017 | SY H R |
|  | LNDG | Northeast, China | Shenyang | 2017 | SY H R |
|  | 82015 | Northeast, China | Shenyang | 2017 | SY H R |
|  | ZF1H | Northeast, China | Shenyang | 2017 | SY H R |
|  | JF1H | Northeast, China | Shenyang | 2017 | SY H R |
|  | CH | Northeast, China | Shenyang | 2017 | SY H R |
|  | ZF2H | Northeast, China | Shenyang | 2017 | SY H R |
|  | 555 | Northeast, China | Shenyang | 2017 | SY H R |
|  | JF2H | Northeast, China | Shenyang | 2017 | SY H R |
|  | FSZ1H | Northeast, China | Shenyang | 2017 | SY H R |
| *C. pinnatifida* var. *major* | HG | East, China | Shenyang | 2017 | SY H R |
|  | XHMZ | East, China | Shenyang | 2017 | SY H R |
|  | MYDJX | East, China | Shenyang | 2017 | SY H R |
|  | BRM | East, China | Shenyang | 2017 | SY H R |
|  | CK | East, China | Shenyang | 2017 | SY H R |
|  | DMQ | East, China | Shenyang | 2017 | SY H R |
|  | XLZR | North, China | Shenyang | 2017 | SY H R |
|  | JD1H | North, China | Shenyang | 2017 | SY H R |
|  | DW | Northeast, China | Shenyang | 2017 | SY H R |
|  | QJX | Northeast, China | Shenyang | 2017 | SY H R |
|  | QYMP | Northeast, China | Shenyang | 2017 | SY H R |
|  | KYRZ | Northeast, China | Shenyang | 2017 | SY H R |
| *C. hupehensis* | HBSZ1H | Central, China | Shenyang | 2017 | SY H R |
|  | HBSZ2H | Central, China | Shenyang | 2017 | SY H R |
|  | HBSZ3H | Central, China | Shenyang | 2017 | SY H R |
|  | MHL | Central, China | Shenyang | 2017 | SY H R |
|  | XPZM | East, China | Shenyang | 2017 | SY H R |
|  | TASS | East, China | Shenyang | 2017 | SY H R |
| *C. pinnatifida* | NMGSLH | North, China | Shenyang | 2017 | SY H R |
|  | WTSSLH | North, China | Shenyang | 2017 | SY H R |
|  | YB8H | Northeast, China | Shenyang | 2017 | SY H R |
|  | YB6H | Northeast, China | Shenyang | 2017 | SY H R |
|  | 1541SLH | Northeast, China | Shenyang | 2017 | SY H R |
|  | YR5H | Northeast, China | Shenyang | 2017 | SY H R |
|  | ZWSLH | Northeast, China | Shenyang | 2017 | SY H R |
|  | GSSZ | Central, China | Shenyang | 2017 | SY H R |
|  | RR5H | Northeast, China | Shenyang | 2017 | SY H R |
|  | RR3H | Northeast, China | Shenyang | 2017 | SY H R |
|  | HLJMDFSLH | Northeast, China | Shenyang | 2017 | SY H R |
|  | CZSLH | Northeast, China | Shenyang | 2017 | SY H R |
|  | LH | Northeast, China | Shenyang | 2017 | SY H R |
|  | HGSLH | East Asia, Korea | Shenyang | 2017 | SY H R |
| *C. maximowiczii* | MSZ1H | Northeast, China | Shenyang | 2017 | SY H R |
|  | MSZ2H | Northeast, China | Shenyang | 2017 | SY H R |
|  | NASZ | Northeast, China | Shenyang | 2017 | SY H R |
|  | MSZ3H | Northeast, China | Shenyang | 2017 | SY H R |
|  | S4 | Russia, Northern Eurasia | Shenyang | 2017 | SY H R |
| *C. sanguinea* | LNSZ1H | Northeast, China | Shenyang | 2017 | SY H R |
|  | LNSZ2H | Northeast, China | Shenyang | 2017 | SY H R |
|  | LNSZ3H | Northeast, China | Shenyang | 2017 | SY H R |
|  | LNSZ4H | Northeast, China | Shenyang | 2017 | SY H R |
| *C.scabrifolia* | YNSZ1H | Southwest, China | Shenyang | 2017 | SY H R |
|  | YNSZ2H | Southwest, China | Shenyang | 2017 | SY H R |
| *C. monogyna* | DZSZ1H | Britain | Shenyang | 2017 | SY H R |
| *C. laevigata* | HHSZ | Britain | Shenyang | 2017 | SY H R |
| *C. cruss-galli* | JJSZ | North America | Shenyang | 2017 | SY H R |

Note：SY H R means Shenyang Hawthorn Repository

All above materials were deposited in National Fruit-tree Germplasm Resourecs - Shenyang Hawthorn Repository and were allowed to be used in this study by the Directors of this Repository.
