# Supplementary material for: Molecular Analysis of Evolution and Origins of Cultivated Hawthorn (Crataegus spp.) and Related Species in China
Source: Front Plant Sci. 2019 Apr 9;10:443. doi: 10.3389/fpls.2019.00443 (PMC6465762; doi:10.3389/fpls.2019.00443)
Supplement: Supplementary file 4 [file Table_2.docx]

| **Table S2 Hawthorn leaf and fruit characteristics** | | | | | | | | | | | | | | | | | |
| --- | --- | --- | --- | --- | --- | --- | --- | --- | --- | --- | --- | --- | --- | --- | --- | --- | --- |
| Taxon | Leaf | | | | | | | | Fruit | | | | | | | | |
|  | Color | Shape | Length  (cm) | Width  (cm) | Margin | | | Lobe | Weight  (g) | Vertical diameter (cm) | Horizontal diameter (cm) | Surface color | Dot quantity | Dot color | Dot size | Surface state | Shape |
| *C. bretschneideri* | 1,2,3 | 2,3 | 9.74±1.19 cd | 9.43±1.06 a | | 1,2 | 2,3 | | 3.41±0.99 bc | 1.60±0.10 bc | 1.70±0.12 b | 3 | 3,4 | 2 | 1,2 | 3 | 1,2 |
| *C. hupehensis* | 1,2 | 1,4 | 9.90±1.73 cd | 7.23±2.18 bcd | | 2,3 | 1,2 | | 3.81±1.39 b | 1.73±0.28 b | 2.01±0.29 a | 2,3 | 2,3 | 2,3 | 2,3 | 2 | 1,2 |
| *C. maximowiczii* | 1,2 | 1,2 | 10.53±2.02 bc | 8.75±1.04 ab | | 2,3 | 1,2 | | 0.69±0.22 d | 0.99±0.07 d | 1.06±0.04 de | 2,3 | 1 | 2 | 1 | 2 | 1 |
| *C. pinnatifida* var. *major* | 2,3 | 1,2,3 | 10.16±0.98 cd | 9.04±1.11 a | | 1,2 | 3,4 | | 6.06±2.76 a | 2.02±0.43 a | 2.21±0.48 a | 1,3 | 3,4 | 2,3 | 2,3 | 3 | 1,3 |
| *C. sanguinea* | 2,3 | 1 | 8.58±0.42 d | 8.25±0.59 abc | | 3 | 1,2 | | 0.75±0.58 d | 1.03±0.36 d | 1.18±0.01 cd | 2,3 | 1 | 2 | 1 | 2 | 1 |
| *C. pinnatifida* | 2,3 | 2,3 | 8.82±0.81 d | 7.91±1.21 abc | | 2 | 3,4 | | 1.59±0.58 cd | 1.45±0.23 c | 1.38±0.15 c | 1,2 | 3,4 | 2,3 | 2,3 | 3 | 1 |
| *C. scabrifolia* | 2 | 4 | 8.50±0.57 d | 6.90±0.28 cd | | 3 | 2 | | 4.12±1.75 b | 1.85±0.25 a | 2.02±0.36 a | 3 | 2 | 2 | 2 | 2 | 2 |
| *C. monogyna* | 3 | 1 | 12.50±2.98 ab | 5.90±0.24 d | | 3 | 3 | | - | - | - | - | - | - | - | - | - |
| *C. laevigata* | 2 | 2 | 6.50±0.19 e | 6.90±0.29 d | | 3 | 4 | | 0.60±0.16 d | 0.96±0.09 d | 0.78±0.01 e | 2 | 1 | 2 | 1 | 3 | 1 |
| *C. cruss-galli* | 3 | 5 | 12.50±0.16 a | 5.90±0.25 d | | 2 | 1 | | - | - | - | - | - | - | - | - | - |

Note: “--” means no data, because the plants of *C. monogyna* and *C. cruss-galli* in National Hawthorn Germplasm Repository are too small to have fruits; Different letters indicate significant differences among the treatments at p <0.05. In leaf color, 1 means light green; 2 means green; 3 means dark green. In leaf shape, 1 means ovate; 2 means wide ovate; 3 means triangle ovate; 4 means rhomboid ovate; 5 means ovate-lanceolate. In leaf margin, 1means wide saw tooth; 2 means thin saw tooth; 3 means blunt round. In leaf lobe 1 means non-dissected; 2 means shallowly dissected; 3 means moderately lobed; 4 means deeply dissected. In fruit surface color, 1means yellow; 2 means orange red; 3 means red; In fruit dot quantity, 1 means very few; 2 means few; 3 means medium; 4 means many. In fruit dot color, 1 means ashen; 2 means golden; 3 means tawny; 4 means brown. In fruit dot size, 1 means small; 2 means medium; 3 means large. In surface state, 1 means rough; 2 means smooth matte; 3 means smooth glossy. In fruit shape, 1 means sub-rounded; 2 means oblate; 3 means squarish.
