# Supplementary material for: Molecular Analysis of Evolution and Origins of Cultivated Hawthorn (Crataegus spp.) and Related Species in China
Source: Front Plant Sci. 2019 Apr 9;10:443. doi: 10.3389/fpls.2019.00443 (PMC6465762; doi:10.3389/fpls.2019.00443)
Supplement: Supplementary file 5 [file Table_3.docx]

**Table S3** nSSR primers used in this study

| Locus | Forward primers  (5' to 3') | S  ( bp ) | Reverse primer  (3' to 5') | S  ( bp ) | Product size ( bp ) | Ta  (℃) |
| --- | --- | --- | --- | --- | --- | --- |
| C_SSR03 | GATCACTTCGGCCATTGTTT | 20 | AAACACGTAGTGTACCACCCG | 21 | 143 | 55 |
| C_SSR04 | AATATTTGACCCGCTGCAAG | 20 | TTCTGCAGGAAAAACCCATC | 20 | 130 | 55 |
| C_SSR09 | GACCACCTCCTTGTCTTCCA | 20 | GTGGGAAAACCAAACCTGAA | 20 | 186 | 56.5 |
| C_SSR17 | TGTATCAGCCTCGACGACAG | 20 | TTCCCCTCCTCCACTTTACC | 20 | 182 | 59 |
| C_SSR19 | CTTGGAAGGTGAGGACGGTA | 20 | CCGGAGGGAAACTAAAACAA | 20 | 278 | 56.5 |
| C_SSR27 | GGTGCGCATCCTTTTGTAAT | 20 | TGGCCGTGTTAAGGTCTTTT | 20 | 151 | 58 |
| C_SSR35 | TGTTCTTGGTCCACTGTTGG | 20 | AAATTCGCCTTTGGTCAAAAT | 21 | 246 | 59 |
| C_SSR37 | ATTCCAGTTCCCAGGAAGGT | 20 | ATTGTTGACTTGTCCCCTGC | 20 | 201 | 59 |
| C_SSR38 | TTTTCCACCGTTAGGAGTCG | 20 | TTAATGGACCGCCATAGAGC | 20 | 218 | 59 |
| C_SSR40 | CTTTCCCAAAAATCAGCGAA | 20 | GGAAGAATTTCGGACGTCAA | 20 | 207 | 58 |
| C_SSR46 | CATCTGCCAACCTTGTTTCA | 20 | AACATCCACACTTGACAGACAAA | 23 | 258 | 58 |
| C_SSR56 | GGTCAGGAGAGAGGATGCTG | 20 | CGCTGAGAGAGAGTGCTTCTT | 21 | 261 | 58 |
| C_SSR58 | AGAAGAAGTGGCGACAGCAT | 20 | AACTCTCTCGAACCGACGAA | 20 | 216 | 58 |
| C_SSR60 | GATCAGATGACACCACACGG | 20 | ATCCAAGCCGTGAGTCAATC | 20 | 120 | 56.5 |
| C_SSR61 | CGCTAGACGCGGTAGAAAAA | 20 | GCAGGGTTTTAGTGGGGACT | 20 | 272 | 58 |
| C_SSR66 | AGCTCTCGATGCCACTCATT | 20 | CGAAGAGCTTCTCAACTGGG | 20 | 230 | 58 |
| C_SSR69 | CAATTGTTTGGGTTTGGGTC | 20 | TATCCGCCATCCTTGACTTC | 20 | 121 | 56.5 |
| C_SSR79 | CACCTTTCCCAATAGACCGA | 20 | CGCTGTCTCTCCTCCATCTC | 20 | 268 | 56.5 |
| C_SSR99 | TCCACACCACCTTCTGATGA | 20 | CTGTCATTTCAATTTCCGTCA | 21 | 268 | 58 |
| C_SSR100 | GATACAACCAGAAAGCCCCA | 20 | GCCAAACTTTCCACACCAAC | 20 | 279 | 58 |
